# UNC-40/DCC, SAX-3/Robo, and VAB-1/Eph Polarize F-Actin during Embryonic Morphogenesis by Regulating the WAVE/SCAR Actin Nucleation Complex

**DOI:** 10.1371/journal.pgen.1002863

**Published:** 2012-08-02

**Authors:** Yelena Y. Bernadskaya, Andre Wallace, Jillian Nguyen, William A. Mohler, Martha C. Soto

**Affiliations:** 1 Department of Pathology and Laboratory Medicine, Robert Wood Johnson Medical School, University of Medicine and Dentistry New Jersey, Piscataway, New Jersey, United States of America; 2 Department of Genetics and Developmental Biology and Center for Cell Analysis and Modeling, University of Connecticut Health Center, Farmington, Connecticut, United States of America; University of California San Diego, United States of America

## Abstract

Many cells in a developing embryo, including neurons and their axons and growth cones, must integrate multiple guidance cues to undergo directed growth and migration. The UNC-6/netrin, SLT-1/slit, and VAB-2/Ephrin guidance cues, and their receptors, UNC-40/DCC, SAX-3/Robo, and VAB-1/Eph, are known to be major regulators of cellular growth and migration. One important area of research is identifying the molecules that interpret this guidance information downstream of the guidance receptors to reorganize the actin cytoskeleton. However, how guidance cues regulate the actin cytoskeleton is not well understood. We report here that UNC-40/DCC, SAX-3/Robo, and VAB-1/Eph differentially regulate the abundance and subcellular localization of the WAVE/SCAR actin nucleation complex and its activator, Rac1/CED-10, in the *Caenorhabditis elegans* embryonic epidermis. Loss of any of these three pathways results in embryos that fail embryonic morphogenesis. Similar defects in epidermal enclosure have been observed when CED-10/Rac1 or the WAVE/SCAR actin nucleation complex are missing during embryonic development in *C. elegans*. Genetic and molecular experiments demonstrate that in fact, these three axonal guidance proteins differentially regulate the levels and membrane enrichment of the WAVE/SCAR complex and its activator, Rac1/CED-10, in the epidermis. Live imaging of filamentous actin (F-actin) in embryos developing in the absence of individual guidance receptors shows that high levels of F-actin are not essential for polarized cell migrations, but that properly polarized distribution of F-actin is essential. These results suggest that proper membrane recruitment and activation of CED-10/Rac1 and of WAVE/SCAR by signals at the plasma membrane result in polarized F-actin that permits directed movements and suggest how multiple guidance cues can result in distinct changes in actin nucleation during morphogenesis.

## Author Summary

Neurons and other cells are most dynamic during embryonic development. Later in life, a diminished developmental potential in mature neurons makes injuries difficult to treat. Neuronal regeneration studies have benefited from genetic studies that identified the molecules that work during embryonic development to give neurons their plasticity and dynamism. Growth and regeneration require dynamic changes in the actin cytoskeleton. In this study we ask how signals that are known to guide migrations of neurons and other cells during development and regeneration are able to reorganize the actin cytoskeleton. Understanding how multiple signals are integrated to control actin dynamics is essential for understanding how cellular movements are regulated in embryos and adults. We find that the actin-nucleation regulating WAVE/SCAR complex molecules, which are conserved from the *C. elegans* soil nematodes to human beings, are localized and regulated by axonal guidance signals during embryonic development. These studies illustrate a mechanism by which different signals reorganize cellular F-actin through their regulation of the actin regulating WAVE/SCAR complex.

## Introduction

Cell migration in response to signals from outside the cell drives developmental processes from embryonic morphogenesis and the establishment of the nervous system, to aberrant migrations during diseases like metastatic cancer. Understanding how cells respond to signals is particularly complicated in developing embryos where tissues, or groups of cells of related identity, must often respond to multiple migration signals while maintaining the integrity of the migrating tissue. It has been proposed that outside signals lead to cellular movements through the rearrangement of the F-actin cytoskeleton. However, the details of how this is accomplished are still being worked out. Ultimately, understanding this process will require understanding how the outside signals are able to organize the cellular cytoskeleton. In this study we addressed what specific changes in the actin cytoskeleton occurred when different migration signals were removed. In addition, we asked if changes in the levels or localization of specific F-actin regulators in response to the migration signals could explain the changes in the actin cytoskeleton and in cell migration.

Studies in *C. elegans* have identified three pathways that guide the migrations of axons during development. *C. elegans* forward genetic screens led to the identification of the netrin/UNC-6 cue that signals to the UNC-40/DCC receptor to guide axonal migrations in larvae [1], [2]. Two additional signaling pathways, ephrin and Robo signaling, guide axonal migrations in *C. elegans* larvae [3]–[6]. In addition, ephrin and Robo signaling contribute to the epidermal cellular migrations that result in epiboly in *C. elegans* embryos. The ephrin VAB-2/EFN-1 and its Eph receptor VAB-1, the only *C. elegans* Eph receptor tyrosine kinase, are required in embryonic neuroblasts to permit epidermal cell enclosure [7], [8]. SAX-3/Robo is essential during embryonic morphogenesis, with requirements within both the migrating epidermis and the underlying neuroblasts for epidermal cell migrations [9]. In contrast, the ligand for SAX-3/Robo, SLT-1, has no embryonic phenotype on its own, suggesting that SAX-3 either has additional ligands besides SLT-1, or does not need a ligand to mediate its embryonic effects [10]. Netrin/UNC-6 and its receptor, UNC-40, have not been examined for epidermal cell migration defects during embryonic development, although neuronal and mesodermal cell migration defects were reported [1]. In addition, tagged UNC-6 and rescuing UNC-40/DCC transgenes are expressed in embryos [2], [11].

Cell migrations in the embryo require dynamic rearrangements of the actin cytoskeleton. Our previous studies have identified an actin nucleation pathway, including the small GTPase CED-10/Rac1, the WAVE/SCAR complex and the Arp2/3 complex, as essential components for embryonic morphogenesis [12]. Mutations or depletion by RNAi of the GTPase CED-10/Rac1, any WAVE/SCAR component, or any Arp2/3 component result in complete loss of epidermal cell shape changes and cell movements. The resulting loss of epidermal cell migration leads to the Gex (gut on the exterior) phenotype first described for WAVE/SCAR complex components GEX-2/Sra1/p140/PIR121/CYFIP and GEX-3/NAP1/HEM2/Kette [12], [13]. The Arp2/3 complex nucleates branched actin polymers, however it is a poor actin nucleator until it is activated by Nucleation Promoting Factors (NPFs) like WAVE/SCAR. The WAVE/SCAR complex is thought to be activated through membrane recruitment by the small GTPase Rac. Of the three *C. elegans* Rac-like GTPases, we have proposed that CED-10/Rac1 functions like the upstream Rac that recruits WAVE/SCAR during embryonic development. We based this proposal on the strong *ced-10* morphogenesis phenotype that is almost as strong as loss of WAVE/SCAR components or Arp2/3 [12], [13]. It is not known which external signals reorganize the actin cytoskeleton through Arp2/3, nor the impact of distinct signals on the actin cytoskeleton. In addition, what happens downstream of Rac signaling is not well understood. Elegant genetic studies in *C. elegans* neurons have identified complex genetic regulation of actin regulators downstream of multiple Rac GTPases [14]. However, as is true in other organisms, the consequences of Rac signaling on the actin cytoskeleton have not been made clear.

Genetic and molecular studies have suggested that axonal guidance pathways may reorganize F-actin through effects on Rac signaling [15]. Genetic studies suggest UNC-40/DCC signals through CED-10/Rac1 in neurons [16] while *in vitro* studies show that Netrin can activate Rac1 through DCC [17]–[19]. Mammalian studies suggest that the unengaged Eph receptor is permissive for Rac activation, while Ephrin activation leads to RhoA activation, Rac inactivation, and actin depolymerization [20], [21]. In Slit-Robo signaling, activation of Robo receptors by SLIT leads to Rac activation [22], [23]. Studies in *Drosophila* suggest that the Robo receptor signals through the Rac GTPase [22], [24].

In this study we show that three guidance pathways, netrin/DCC, SLIT/Robo and ephrin, regulate embryonic morphogenesis and F-actin polarization through effects on the WAVE/SCAR complex. The receptors in all three pathways are required for the proper levels and organization of F-actin in the embryonic epidermis. All three regulate the subcellular distribution and levels of molecules that are essential for embryonic morphogenesis: CED-10/Rac1, the WAVE/SCAR complex and F-actin. Since each of the three guidance pathways has embryonic morphogenesis defects similar to but milder than the loss of CED-10, WAVE/SCAR or Arp2/3, we tested our hypothesis that the three cues are redundant for a shared morphogenesis function. We found instead that each cue promoted morphogenesis through distinct effects on CED-10/Rac1, WAVE/SCAR and on epidermal F-actin. UNC-40/DCC regulated the membrane enrichment of CED-10, and promoted correct WVE-1 subcellular distribution required for normal levels of F-actin nucleation, but polarization of F-actin was minimally affected. Thus signals from UNC-40/DCC are important for total F-actin levels, and less important for F-actin polarization required for migrations. This essential role of netrin signaling in embryos had probably been missed due to the low penetrance of the phenotypes. SAX-3 positively regulated CED-10/Rac subcellular distribution, which led to WAVE/SCAR recruitment to membranes, and resulted in appropriate levels and polarization of F-actin in migrating cells. Finally, Eph signaling regulated CED-10 distribution, which is required for the correct WAVE complex localization in the epidermis, resulting in properly polarized F-actin in the migrating epidermal cells. This shows that ephrin receptor strongly affects events in the epidermal cells required for polarized F-actin distribution. Thus, these studies illustrate that distinct signals at the plasma membrane result in differential effects on the F-actin regulating WAVE/SCAR complex, required for both the correct levels and polarization of the actin cytoskeleton during morphogenesis. Interestingly, the degree of F-actin polarization, rather than the total levels, seems to determine whether a cell can initiate migrations or not.

## Results

### Axonal guidance receptors are required for embryonic epidermal morphogenesis

**Figure 1:**
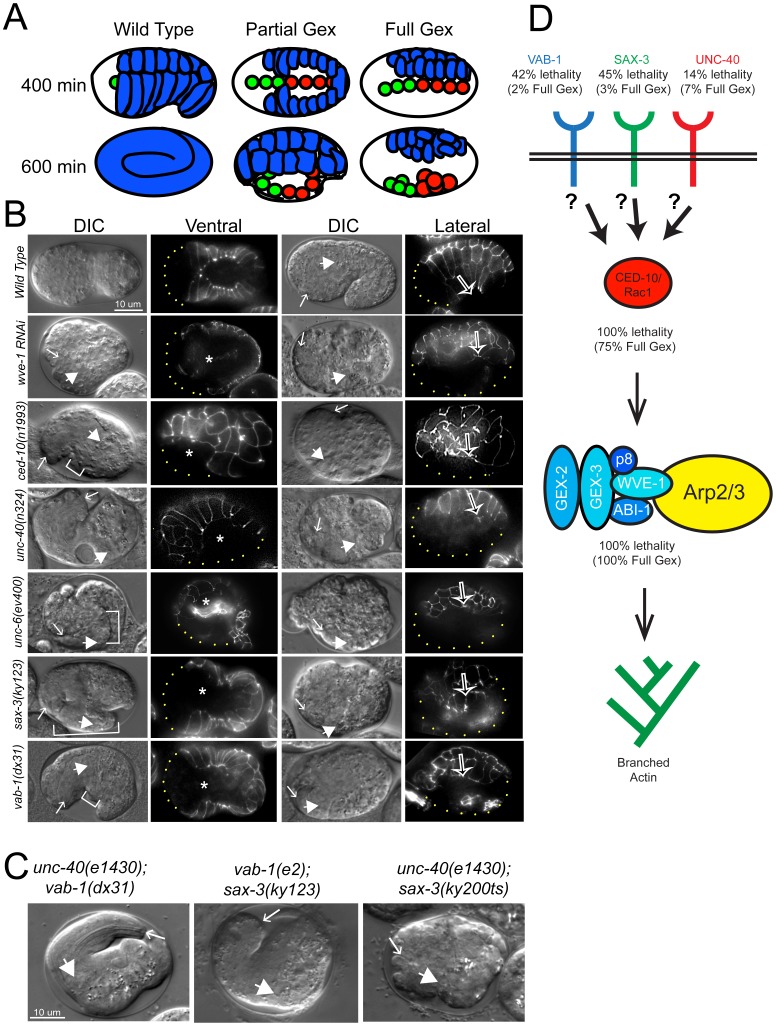
Axonal guidance receptors are required for embryonic epidermal morphogenesis. (A) Comparison of morphogenesis in embryos with wild type, Partial Gex or Full Gex phenotypes. In wild type the epidermal cells (blue) enclose the embryo by ∼400 minutes after first cleavage at 20°C. By ∼600 minutes the embryos elongate to the three-fold stage. In Partial Gex embryos epidermal cells do not fully enclose the embryo by 400 minutes, and by 600 minutes the internal organs (green = pharynx, red = intestine) partially extrude through epidermal gaps. In Full Gex embryos the epidermal cells completely fail cell migration and by 600 minutes the internal organs are fully extruded to the surface of the embryo. (B) Embryonic morphogenesis defects visualized using DIC optics and the *dlg-1::gfp* (*xnIs16*) [25] transgene that marks junctions of epithelial tissues. Embryos are oriented with anterior to the left. Representative images from the ventral (left columns) or lateral (right columns) view and corresponding ventral and lateral images of DLG-1::GFP are shown. *DIC images*. Arrows: anterior pharynx. Block arrows: anterior intestine. White brackets: extruded internal organs. *DLG-1::GFP images*. Asterisks: ventral gap between epidermal cells. Open arrows in right panels: leading edge of epidermis. Dots outline the unenclosed regions of embryos. Alleles shown are putative nulls, including deletion mutations in *ced-10*, *unc-6*, *sax-3* and *vab-1*, with the exception of *ced-10(n1993)* a hypomorph [1], [3], [8], [50]. (C) Morphogenesis failure in genetic doubles of axonal guidance mutants. Labeled as in (B). Most *unc-40(e1430); vab-1(dx31)* and *vab-1(e2); sax-3(ky123)* doubles die with the Partial Gex phenotype. *unc-40(e1430); sax-3(ky200ts)* doubles show a synergistic increase in the number of Full Gex embryos. (D) Summary of the proposed contribution of the axonal guidance receptors, the CED-10/Rac1 GTPase, the WAVE/SCAR complex and the Arp2/3 complex to embryonic viability (% lethality) and epidermal morphogenesis (% Full Gex).

In order to identify the upstream signals that reorganize the actin cytoskeleton during embryonic morphogenesis, we took a candidate gene approach by searching for signaling receptors which shared loss-of-function phenotypes with mutations in the WAVE/SCAR complex, a major regulator of embryonic F-actin organization. *C. elegans* receptors that transmit signals upstream of embryonic actin regulators would be expected to be expressed in embryos, and their loss of function phenotype should include embryos with the Full Gex, or gut on the exterior phenotype seen 100% of the time when WAVE/SCAR or Arp2/3 components are missing (Figure 1A). They might also share the Gex post-embryonic phenotypes including the Egl or Egg laying defective phenotype [13].

#### Embryonic morphogenesis defects of UNC-6/Netrin and UNC-40/Netrin receptor mutants

The UNC-40/DCC receptor came to our attention as a candidate Gex receptor due to its 60% Egl and 100% Dpy (Dumpy) phenotypes. Some Dpy mutants have altered body shape due to cytoskeletal and morphogenesis defects. However, *unc-40* mutants had not been examined for their embryonic epidermal phenotypes. Four different putative null alleles of *unc-40 (e1430, n324, n473, e271*), that are caused by missense mutations predicted to result in truncated UNC-40, and a deletion mutation in the UNC-40 ligand, *unc-6/netrin (ev400)* result in similar levels of embryonic lethality due to failure in morphogenesis (Figure 1B, Table 1). This was true even after all these independently obtained *unc-40* alleles and the *unc-6* deletion null allele were out-crossed to wild type twice to see if the lethality was caused by background differences or secondary mutations (Table 1). For example, *unc-40(n324)* and *unc-6(ev400)* embryos displayed complete arrest of the epidermal migrations, referred to here as the Full Gex morphogenesis phenotype, 7% and 3% of the time (Figure 1B, Table 1). In addition, another 7% of *unc-40* and *unc-6* embryos undergo partial enclosure of the epidermis (referred to here as the Partial Gex phenotype (Figure 1A, 1B; Table 1). To better image the changes in cell movements in live embryos these mutations were crossed into a *dlg-1::gfp* transgene *(xnIs16)* [25] that visualizes cell-cell junctions in epithelia. *unc-40* and *unc-6* embryos were observed to arrest the epidermal migrations at different stages resulting in the Full and Partial Gex phenotypes (Figure 1B). Thus, both Netrin/UNC-6 and its receptor UNC-40/DCC are required for epidermal morphogenetic movements.

**Table 1:** Distribution of embryonic morphogenesis phenotypes.

Genotype	% Wild-type	% Full Gex	% Partial Gex	% Total Lethality	n =
*Wild Type (N2)*	>99	0	<1	<1	1000+
*wve-1(zu496)*	0	100***	0	100***	308
*unc-40(e271)*	93	3	4	7	521
*unc-40(e473)*	87	6	6	13**	93
*unc-40(e1430)*	91	2	7*	9**	554
*unc-40(n324)*	86	7*	7*	14***	503
*unc-6(ev400)*	89	3	7*	10	394
*sax-3(ky123)*	55	3	42*	45***	566
*vab-1(dx31)*	58	2	40*	42***	491
*sax-3(ky200ts) (20°C)*	99	0	1	1	488
*sax-3(ky200ts) (25.5°C)*	85	3	8	12	384
*unc-40(e1430) (20°C)*	96	1	2	3	197
*unc-40(e1430) (25.5°C)*	98	1	1	2	97
*unc-40(e1430); sax-3(ky200ts) (20°C)*	69	9	22	31	378
*unc-40(e1430); sax-3(ky200ts) (25.5°C)*	47	19*	34***	53***	305
*vab-1(e2)*	80	0	20	20	171
*vab-1(e2); sax-3(ky123)*	0	2	98****	100****	50
*unc-40(e1430); vab-1(dx31)*	16	6	78****	84****	299

Distribution of Full and Partial Gex phenotypes observed in single and double genetic mutants. All strains were cultivated at 20°C unless otherwise indicated. Statistically significant changes in phenotype relative to wild type for single mutants, or relative to the two single mutants for double mutants are indicated, calculated by a One-Way ANOVA test followed by the Tukey test.

*:  = p<0.05.

**:  = p<0.01.

***:  = p<0.001,

****:  = p<0.0001.

The finding that UNC-6/netrin and its receptor UNC-40/DCC are involved in embryonic epidermal cell migrations led us to ask which other regulators of neuronal migrations might be contributing to this process. Both SLT-1/Robo and Ephrin pathways are known to affect embryonic morphogenesis in *C. elegans* [26]. We therefore compared the phenotypes of mutations in WAVE/SCAR to mutations in the receptors of these two additional guidance pathways: *sax-3/Robo* and *vab-1/Eph*. As previously reported, *sax-3(ky123)* and *vab-1(dx31*), two deletion null alleles, lead to embryonic lethality in approximately half (45% and 42% respectively) of the embryos [3], [8] (Figure 1, Table 1). Via DIC optics and by crossing *sax-3(ky123)* and *vab-1(dx31*) into the *dlg-1::gfp* strain we observed that 3% of *sax-3* and 2% of *vab-1* embryos display the Full Gex phenotype, while the majority of the dying embryos, 42% and 40% respectively, display a partial enclosure phenotype, with frequent failures in head enclosure (11% and 10%) and in pocket cell enclosure (32% and 30%, Figure 1B brackets, Table 1). Therefore these three different guidance pathways contribute to embryonic morphogenesis.

### Genetic analysis of the contribution of the three guidance cues to embryonic epidermal morphogenesis

The discovery that three signaling pathways, Netrin, Robo and Ephrin, were contributing to embryonic morphogenesis, but with weaker embryonic morphogenesis phenotypes than loss of WAVE/SCAR, led us to use double mutant analysis to test the hypothesis that these cues act redundantly during embryonic morphogenesis. This experiment was complicated by the fact that some double mutant combinations using null alleles are lethal [9] (our unpublished observations). We therefore sometimes used combinations of one null allele and one hypomorphic allele. We predicted that if the pathways are redundant, then removing or reducing two pathways that activated WAVE/SCAR would lead to more penetrant Full Gex phenotypes. We found that all the double mutant combinations led to increased embryonic lethality, and synergistic increases in the Partial Gex lethality. However only the *unc-40; sax-3* double mutant displayed a synergistic increase in the Full Gex phenotype (Table 1). The double mutants between the *vab-1* null allele, *dx31* (2% Full Gex) and the putative null allele *unc-40(e1430)* (3% Full Gex) or between the *vab-1* hypomorph, *e2* (0% Full Gex), and the *sax-3* null allele, *ky123*, (3% Full Gex) resulted in only 6% and 2% Full Gex, respectively (Table 1, Figure 1C). These experiments did not support simple redundancy between the *vab-1* pathway and either the *unc-40* or *sax-3* pathway for the regulation of the WAVE complex and are better explained by the pathways having parallel inputs that result in the Gex phenotype. The double between the putative null *unc-40(e1430)* (1% Full Gex *at* 25°C) and the hypomorphic *sax-3(ky200ts)* allele (9% Full Gex at 25°C) at the restrictive temperature (25°C) increased the Full Gex phenotype to 19% (Table 1, Figure 1C). This synergistic effect on the Full Gex phenotype suggested that there is redundancy between the *unc-40* and *sax-3* pathways for activating the WAVE complex.

### Guidance pathway proteins regulate F-actin organization and levels in migrating embryonic cells

**Figure 2:**
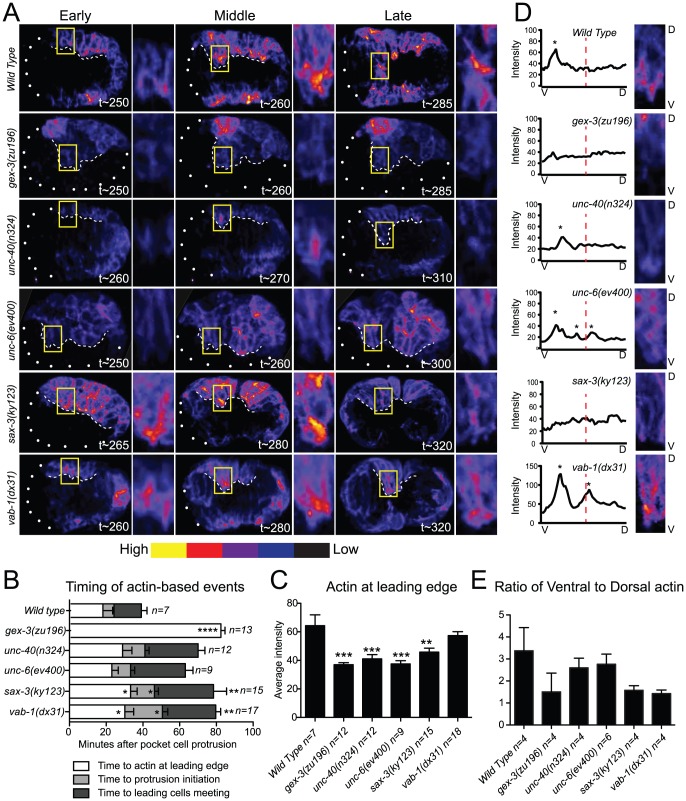
Guidance pathway proteins regulate F-actin organization and levels in migrating embryonic cells. Embryos are oriented with anterior to the left. (A) Polymerized actin visualized in 4D using the *plin26::vab10ActinBindingDomain::gfp* transgene (*mcIs51*) [12], [27]. Embryos imaged at 2-minute intervals for 2 hours, beginning at 240 minutes, at 23°C. Embryos at Early, Middle, and Late stages, as related to F-actin-dependent events (B), are shown. t = minutes after first cleavage. Dots outline unenclosed regions of the embryo. Leading Cells (LCs) are boxed and magnified. White dashed line: leading edge. Embryos were pseudo-colored using GLOWormJ “Fire” setting, from low (blue) to high (yellow) intensity. (B) Time intervals between actin-dependent events. Time “0” corresponds to the first appearance of epidermal pocket cells protrusions, at approximately 250 minutes after first cleavage in wild type and mutants. (C) The intensity of F-actin in the LCs analyzed at the time of actin enrichment at the leading edge. The two LCs (yellow boxed region shown in A) were compared. Additional details about F-actin measurements in Materials and Methods. (D). Distribution of actin peaks in the ventral half compared with the dorsal half of LCs. Close-ups of representative embryos at Late stages (∼290 min.) are shown. V = Ventral, bottom. D = Dorsal, top. A ventral to dorsal line was drawn through the cell using the Plot Profile tool in GLOWormJ and fluorescent intensity was measured. Peaks, defined as regions at least 10 fluorescent units higher than the background, were counted and are marked by asterisks. Dashed red lines mark half the cell's length. (E) Ratios of ventral to dorsal actin distribution based on the actin peaks measured as in D during 40 minutes beginning with the enrichment of actin at the leading edge. Error bars show SEM. Asterisks mark statistical significance, * = p<.05, *** = p<0.001 as determined by a One-way Anova test followed by the Tukey test.

Since loss of the guidance pathway proteins altered morphogenetic movements, these proteins may be required for the organization of the actin cytoskeleton. We therefore analyzed the effects of the three guidance pathways on the *plin-26::vab-10 ABD (actin binding domain)::gfp (mcIs51)* transgene that permits live imaging of F-actin enrichment in the epidermis of embryos undergoing morphogenesis [12], [27]. In wild-type embryos this transgene is expressed at high levels in the six rows of epidermal cells. Wild-type embryos have abundant F-actin that is dynamically enriched toward the leading edge of the ventral migrating cells. In particular, the two anterior Leading Cells (LCs) on each side, which are essential for guiding the ventral migration of the epidermis [28], make large filopodial protrusions and form a broad lamellar front (Figure 2A). The highest F-actin enrichment was seen, on average, 2 µm behind the protrusive front in the LCs. Loss of either *arp-2* or *gex-3* resulted in less actin at the leading edge and loss of filopodial protrusions and lamellar protrusions in the LCs [12] (Figure 2A, 2C, Videos S1 and S2).

To compare the F-actin organization in wild-type, WAVE/SCAR depleted, and guidance pathway mutants, we made movies starting at 240 minutes after first cleavage, when epidermal morphogenesis begins in wild type, and collected images every 2 minutes until at least 380 minutes after first cleavage at 23°C (see Materials and Methods). Since *sax-3* and *vab-1* mutants have delayed gastrulation movements that precede epidermal migration, we timed the occurrence of actin-based events. The time intervals between first large protrusions of the pocket cells, ventral actin enrichment in the Leading Cells (LCs), LC protrusion initiation, and ventral meeting of the LCs were increased in *unc-40*, *sax-3*, and *vab-1* mutants (Figure 2B), and are reminiscent of the gastrulation cleft closure delays described for *sax-3* and *vab-1* [8], [9]. We therefore used the first protrusion of the pocket cells, which occurs at 250 minutes in wild-type and mutant embryos, and the meeting of the LCs, which occurs at 320 minutes in wild-type embryos, but later in mutant embryos, as reference points, and measured actin accumulation relative to these events (Figure 2B). Crossing the *plin-26::vab-10 ABD::gfp* transgene into animals carrying putative null alleles of *unc-40, unc-6, sax-3* and *vab-1* illustrated the distinct effects of each axonal guidance pathway on epidermal F-actin.

**Figure 3:**
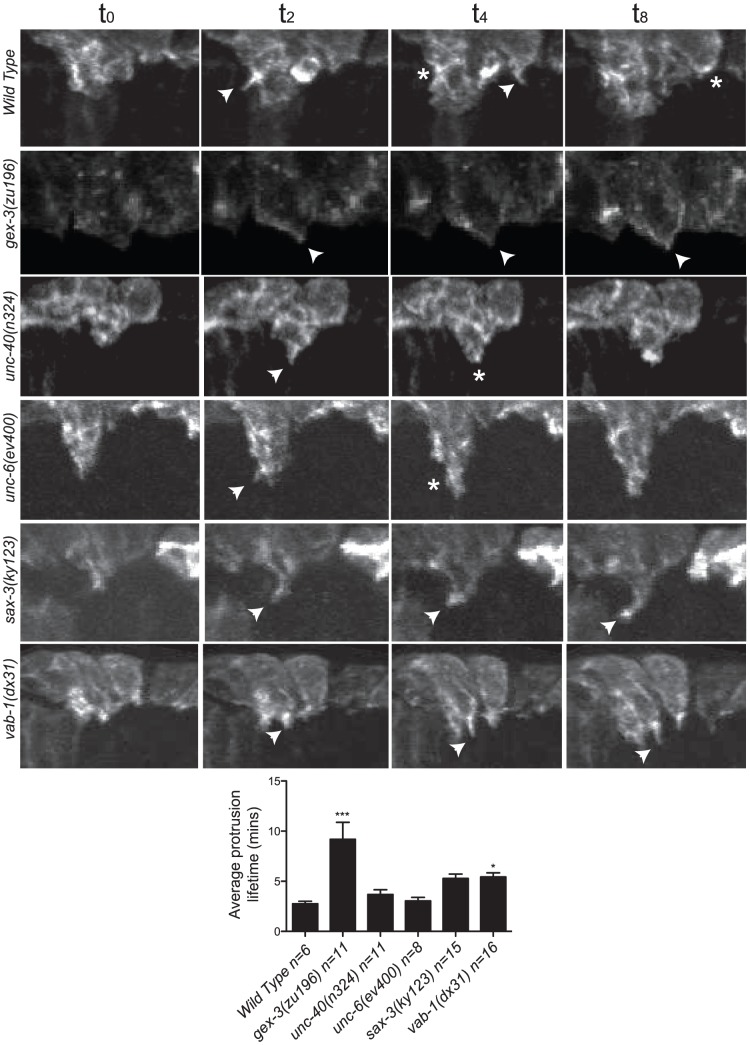
The dynamic turnover of F-actin protrusions is altered in morphogenesis mutants. F-actin protrusions produced by the two Leading Cells (LCs) on one side during epidermal cell migration were analyzed using the *plin26::vab10 Actin Binding Domain::gfp* transgene (*mcIs51*) [12], [27]. Micrographs are close-ups of migrating LCs. Arrows point to protrusions. Asterisks denote site of protrusion retraction. Four consecutive time points are shown with an arbitrary timing of t0 set as 2 minutes before protrusion formation. Bar graph shows the average duration of the F-actin protrusions in minutes. Error bars show SEM. Asterisks mark statistical significance, * = p<.05, *** = p<0.001 as determined by a One-way Anova test followed by the Tukey test.

Most embryos missing *unc-40* or *unc-6* are able to enclose and live (Figure 1B, Table 1). Loss of *unc-40* or *unc-6* resulted in a significant decrease of overall F-actin levels in the ventral row of migrating epidermal cells for all time points observed, but undiminished protrusive activity. In the *unc-40* and *unc-6* embryos shown in Figure 2A all the ventral cells express low levels of F-actin, yet the LCs are still capable of protrusive activity including filopodial protrusions (Videos S1 and S2, Figure 2A, Figure 3). Further, the distribution of F-actin from ventral to dorsal regions of the LCs showed similar ventralward enrichment as seen in wild-type embryos (Figure 2D, 2E). The continued dynamic protrusions and the ventral F-actin enrichment explain the relatively mild embryonic lethality of *unc-40* and *unc-6* embryos.

Close to half of the embryos missing *sax-3* (45%) fail to enclose and die (Figure 1B, 1D, Table 1). On average, *sax-3* embryos had decreased ventral F-actin levels by 330 minutes (Figure 2A, 2C, Videos S1 and S2). These embryos were delayed in initiating ventralward movements (Figure 2B), especially in the LCls (Figure 1B, Figure 2A), and show decreased ventralward enrichment of F-actin (Figure 2D, 2E).

Close to half of the embryos missing *vab-1* (42%) fail to enclose and die (Figure 1, Table 1). Loss of *vab-1* resulted in increased levels of epidermal F-actin in the ventral-most row of epidermal cells by 300 minutes (Figure 2A), but disorganization of the F-actin distribution (Figure 2E). *vab-1* mutant embryos maintained high F-actin levels at the leading edge (Figure 2C), but had inappropriately increased enrichment of F-actin on the dorsal side of the LCs (Figure 2A, 2D, 2E). The LCs were delayed in making filopodia or lamella compared to wild type (Figure 2A, 2B, Videos S1 and S2). Thus ephrin signaling supports ventrally enriched F-actin distribution that correlates with the timely migration of the epidermal cells and with successful enclosure.

### The dynamic turnover of F-actin protrusions is altered in morphogenesis mutants

The F-actin movies were captured at 2-minute intervals to minimize phototoxicity, so we could not measure rapid changes in actin dynamics. However, the movies show that in wild type, protrusions at the leading edge form and retract dynamically, on average lasting 2.5 minutes. These dynamic changes contribute to the ventralward migration of the cells. In contrast, in the WAVE/SCAR and axonal guidance mutants, protrusions often formed and retracted more slowly, leading to minimal changes at the leading edge. In wild type, *unc-40* or *unc-6* embryos, protrusions lasted, on average, 2.5 to 3 minutes, and the majority of embryos enclosed. In contrast in *gex-3*, *sax-3* and *vab-1* embryos, protrusions lasted 9, 6 and 6 minutes, respectively, and 100%, 45% and 42% of the embryos failed to enclose (Figure 3).

### Guidance receptors affect subcellular distribution of Rac1/CED-10

#### Rac1/CED-10 regulates the levels and subcellular distribution of WVE-1

Since guidance receptor mutants shared phenotypes with CED-10 and WAVE/SCAR mutants, we decided to test if the guidance cues directly affected CED-10/Rac1 and WAVE/SCAR. Cell surface receptors are proposed to activate CED-10/Rac1, and GTP-bound Rac1 is able to bind to the WAVE/SCAR component GEX-2/Sra1/p140/PIR121/CYFIP, which in turn recruits the rest of the WAVE complex to the membrane [29]–[31]. We first tested if loss of *ced-10* altered WVE-1 levels or distribution and found changes in both. The *ced-10(n1993)* hypomorphic allele contains a mutation in a prenylation site predicted to bring CED-10 to the plasma membrane [32]. The *ced-10(n1993)* mutation reduced the apparent GFP::WVE-1 levels in 26 of 28 embryos examined (Figure 5A) and reduced GFP::WVE-1 enrichment relative to the basolateral protein UNC-70/beta spectrin [33] (Figure 5B). Whole worm or embryonic *ced-10(n1993)* lysates (Materials and Methods) contain lower levels of endogenous WVE-1 (Figure 5C). Performing subcellular fractionations (Materials and Methods) we previously showed that the low speed P1 pellet is enriched in plasma membrane proteins, including PH::GFP, and in large membranous organelles like the Golgi. We proposed that pellet enrichment reflects membrane association of proteins [34]. Control lysates showed that in wild-type animals the low speed pellet (P1) contains 24% of endogenous WVE-1. Fractionated *ced-10(n1993)* lysates showed large increases in WVE-1 enrichment in the P1 and P2 pellets (Figure 5E), suggesting that CED-10 regulates the recruitment of WVE-1 to the correct membranes.

#### Effects of receptor mutants on CED-10::GFP

**Figure 4:**
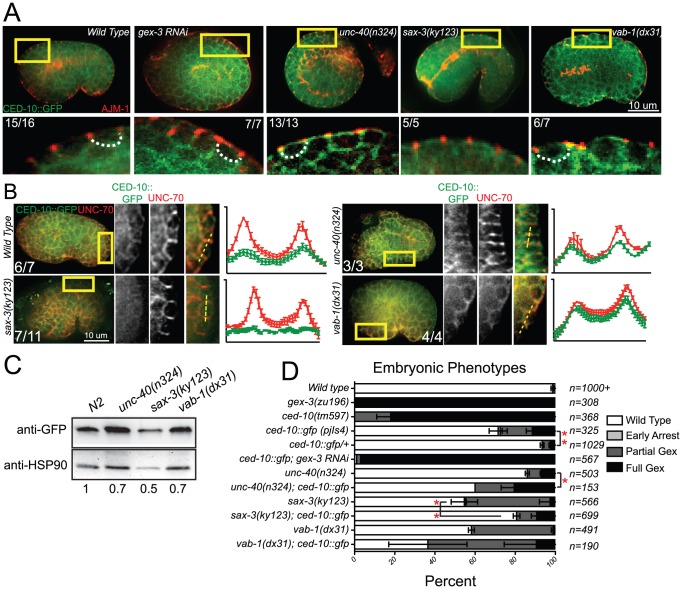
Guidance receptors affect subcellular distribution and levels of the GTPase CED-10/Rac1. All embryos are oriented with anterior to the left and dorsal up. Boxed regions are amplified and enhanced equally for contrast. Number of embryos that showed the represented phenotype are indicated. (A) Embryos carrying an integrated rescuing *ced-10::gfp* (*pjIs4*) transgene were double-stained with antibodies to GFP (Abcam, ab6556) and to endogenous AJM-1 to indicate the adherens junctions [51], [52]. Dotted lines outline the basolateral region of the epidermal cells if the region is discernable. (B) Embryos carrying an integrated rescuing *ced-10::gfp* transgene were double stained with antibodies to GFP [49] and to endogenous basolaterally localized UNC-70/beta spectrin [33]. Readings were taken across two cell junctions using the line tool in ImageJ for both the UNC-70 and the GFP signal. 5 readings were taken per cell and averaged, and plotted in IPad Prism. SEM is shown. (C) Total levels of CED-10::GFP measured with antibody to GFP (Abcam, ab6556). Numbers below each lane are the levels of GFP normalized to HSP90 (Abcam ab13492) as loading control, and relative to wild type, averaged from 4 blots from two sets of lysates. (D) Genetic interactions of WAVE/SCAR and guidance receptor mutants with the integrated rescuing CED-10::GFP transgene. “Early Arrest” refers to embryonic arrest before morphogenesis begins. Asterisks indicate significant change in the phenotypes compared to the single mutants. * = p<.05, *** = p<0.001 as determined by a One-way Anova test followed by the Tukey test.

Given the clear connection between Rac1/CED-10 and the levels and localization of WVE-1, we analyzed the effects of the three guidance pathways on the subcellular distribution and levels of CED-10 utilizing the rescuing transgene, *ced-10::gfp* [35], which we integrated into the genome using UV-TMP. In wild-type embryos undergoing epidermal cell migrations this transgene shows a similar basolaterally enriched epidermal expression pattern, basal to the apical junction component AJM-1 (Figure 4A), as we have reported for F-actin and for other WAVE/SCAR components, GEX-2 and GFP::GEX-3 [12], [13]. The dorsal epidermal cells highlighted in Figure 4 are undergoing convergent extension-like dorsal intercalation movements that require the WAVE/SCAR complex [12], [13]. CED-10::GFP membrane enrichment was reduced in *sax-3* null mutants, and appeared increased and disorganized in *unc-40* and *vab-1* null mutants. Loss of *unc-40* or *vab-1* resulted in ectopic CED-10::GFP at apical membranes (Figure 4A). Double labeling of CED-10::GFP and the basolateral marker UNC-70/beta spectrin showed that in *sax-3* embryos less CED-10::GFP localizes to basolateral regions of these epidermal cells, while in *unc-40* and *vab-1* embryos relatively more CED-10::GFP appears enriched at the basolateral regions. These results suggested that the guidance molecules regulate the subcellular distribution of CED-10. To test if the axonal receptors also affect the total levels of CED-10, we made whole worm lysates (Materials and Methods) from the CED-10::GFP strain carrying null mutations in *unc-40, sax-3* or *vab-1* and probed western blots with an antibody to GFP. Loss of the three guidance molecules, especially *sax-3*, reduced total CED-10::GFP levels (Figure 4C).

#### Genetic interactions between receptor mutants and CED-10::GFP

The integrated, rescuing CED-10::GFP strain was made from an extrachromosomal array, thus is likely to contain multiple copies of the wild type *ced-10* gene. This strain on its own has some Full Gex lethality, suggesting too much wild type CED-10 interferes with morphogenesis. Increasing any Rho GTPase can saturate the cellular machinery that regulates the balance of Rho, Rac and CDC42 [36], [37]. Extra copies of CED-10 may saturate molecules like RHI-1/RHOGDI (Rho guanine nucleotide dissociation inhibitor), and thus be deleterious by interfering with the other GTPases in the cell. To test if additional CED-10 causes lethality we compared the progeny of animals that were homozygous or heterozygous for the integrated CED-10::GFP transgene and found that the embryonic lethality was dose dependent: embryonic lethality was twice as high in the progeny of homozygotes vs. heterozygotes (Figure 4D). We therefore asked how the integrated homozygous GFP::CED-10 array affected animals that were also missing *unc-40*, *sax-3* or *vab-1*. CED-10::GFP significantly increased the Full Gex phenotype in *unc-40(n324)* and increased the Partial Gex lethality in *vab-1(dx31)*. CED-10::GFP significantly decreased the overall embryonic lethality and the percent of embryos with the Partial Gex phenotype in *sax-3(ky123)* animals (Figure 4D). This result suggested additional copies of CED-10 can rescue some *sax-3* defects, while worsening *unc-40* and *vab-1* defects.

### Guidance receptors affect subcellular distribution and levels of WVE-1

#### Effects of receptor mutants on GFP::WVE-1

**Figure 5:**
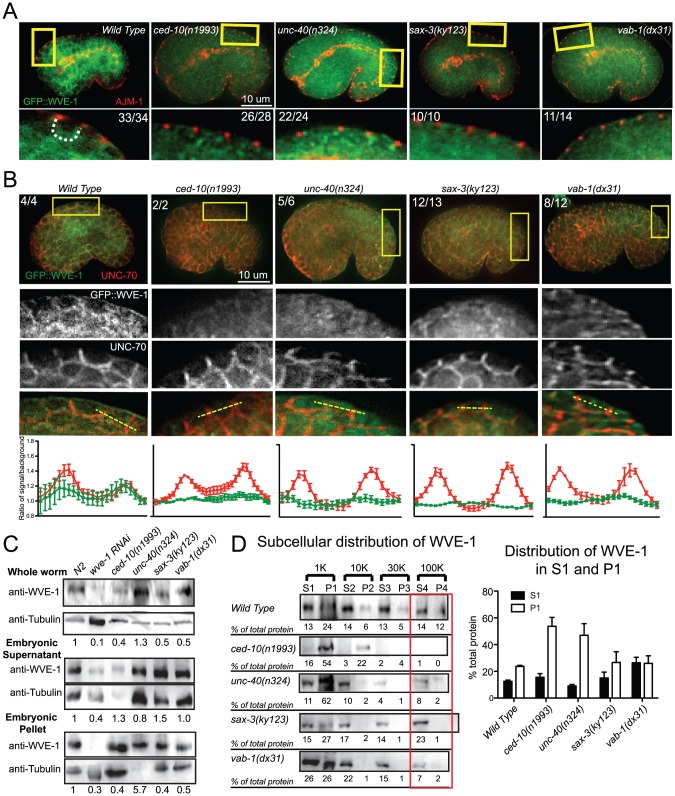
Guidance receptors affect subcellular distribution and levels of WAVE/SCAR. Embryos are oriented with anterior to the left and dorsal up. (A) Embryos carrying the integrated, rescuing *gfp::wve-1* (*pjIs1*) transgene were double-stained with antibodies to GFP (Abcam, ab6556, polyclonal) and AJM-1. Boxed regions are amplified and enhanced equally for contrast. Number of embryos that showed the represented phenotype are indicated. Dotted line outlines the basolateral region of the epidermal cells where the region is discernable. (B) Embryos carrying the rescuing integrated *gfp::wve-1* transgene were double stained with mAb to GFP [49] and basolaterally localized UNC-70/beta spectrin [33]. Readings were taken across two cell junctions using the line tool in ImageJ for both the UNC-70 and the GFP signal. 5 readings were taken per cell and averaged and plotted in IPad Prism. SEM is shown. (C) Total levels of endogenous WVE-1 in whole worm lysates, or embryonic lysates (Materials and Methods) measured with a polyclonal antibody to WVE-1 [12]. Levels of WVE-1 normalized to tubulin and relative to WT are shown below the graph, based on the average of 4 blots from 3 sets of lysates. (D) Subcellular distribution of WVE-1 in fractionated lysates, measured using an antibody to endogenous WVE-1. Lysates were spun at increasing speeds and duration. Pellets were resuspended to match the volume of their partner Supernatant fraction. Equal volumes of each S and P fraction were loaded so that relative amounts of protein in the S vs. P fraction could be compared [34] (See Materials and Methods). 10 µl of each fraction were loaded. Numbers below each band represent the relative percentage of total protein found in each fraction. Numbers represent average of three blots (one set of lysates). S = supernatant, P = pellet. Graph shows average of total protein in S1 and P1 based on 3 blots. SEM is shown. Red rectangles indicate fractions with most significant changes compared to WT.

Since the signaling molecules appeared to regulate CED-10 subcellular localization (Figure 4) and since CED-10 regulates WAVE levels and localization (Figure 5), we next wanted to examine how the signaling molecules affected WAVE.

To monitor the effect of the candidate upstream signals on the WAVE complex we generated a GFP-tagged WVE-1 transgene that rescues putative null mutations in *wve-1* and integrated it into the genome using UV-TMP [38]. In wild-type embryos this transgene shows enriched GFP::WVE-1 at regions of cell-cell contact, including the basolateral epidermis [12] (Figure 5A, 5B). When the rescuing GFP::WVE-1 transgene (*pjIs1*) was crossed into animals with null mutations in *unc-40*, *sax-3* or *vab-1*, all three led to reduced accumulation of GFP::WVE-1 at the basolateral epidermis. This altered accumulation was seen in embryos doubly labeled for GFP::WVE-1, using an anti-GFP Rabbit antibody, and the junctional marker AJM-1 (Figure 5A) and in embryos doubly labeled for GFP::WVE-1, using a mouse anti-GFP antibody, and the antibody to the basolateral marker UNC-70/beta spectrin. The effect on basolateral GFP::WVE-1 enrichment by loss of the signaling molecules was similar to that caused by loss of *ced-10(n1993)*. These results suggested two possible effects of the guidance receptors on WVE-1: changes in the overall levels and changes in the subcellular localization.

#### Effects of receptor mutants on total WVE-1 levels

To directly measure the levels of WVE-1 in the guidance mutants, we made lysates from animals carrying null mutations in *unc-40*, *sax-3* and *vab-1*, and probed western blots with an antibody to *C. elegans* WVE-1 [12]. We show the results for whole worm lysates (Materials and Methods), which include WVE-1 found in pellets and supernatants, since the fractionation studies (Figure 5D) revealed dramatic shifts in WVE-1 when the upstream signals are removed. Control lysates in which *wve-1* was depleted by RNAi resulted in approximately 90% loss in the levels of WVE-1. Lysates missing UNC-40 showed increased total levels of WVE-1. Lysates missing SAX-3 or VAB-1 showed a decrease in the overall level of WVE-1 (Figure 5C). These results showed that in agreement with the results from live imaging with a rescuing GFP::WVE-1 transgene, loss of SAX-3 or VAB-1 resulted in decreased levels of WVE-1 protein.

#### Effects of receptor mutants on membrane localization of WVE-1

To test if the altered distribution of WVE-1 resulted from a shift of WVE-1 enrichment in membranes or cytoplasm, we used control lysates and lysates made from animals carrying null mutations in *unc-40*, *sax-3* and *vab-1* to perform subcellular fractionations. Loss of *unc-40*, like the prenylation-defective *ced-10(n1993)* lysates, resulted in a dramatic increase in the amount of WVE-1 in the low speed, P1 pellet (62%), at the expense of other pellets and supernatant fractions (Figure 5D). This result suggests that loss of UNC-40 leads to more WVE-1 enrichment at membranes and organelles enriched in the P1 pellet. Loss of SAX-3 or VAB-1 did not significantly affect WVE-1 distribution in the low speed P1 pellet. The strongest effect observed was decreased WVE-1 in the high speed P3 and P4 fractions and a shift into supernatant fractions (Figure 5D). We previously showed that P4 is enriched for transmembrane proteins of the recycling endosome while S4 is enriched for membrane-associated proteins of the recycling endosome [34]. Therefore the loss of SAX-3 and VAB-1 may influence WVE-1 distribution at recycling endosomes or other endosomal fractions. Loss of SAX-3 or VAB-1 disrupts WVE-1 association with multiple membranes.

In summary, the three pathways differentially regulate the overall levels of WVE-1 (Figure 5C), while all three pathways affect the subcellular distribution of WVE-1 (Figure 5A, 5B, 5D). The consequences of this regulation can be appreciated by *in vivo* imaging, which reveals reduced basolateral WVE-1 enrichment in epidermal cells undergoing morphogenesis (Figure 5B). In *sax-3* mutants this reduced membrane enrichment is coupled to decreased levels of WVE-1. The lower levels of WAVE/SCAR measured in *sax-3* embryos may contribute to the lower ventral F-actin levels in these embryos (Figure 2C). In *unc-40* mutants, WVE-1 membrane levels increase, but WVE-1 may not be properly polarized to basolateral regions as it appears more uniformly distributed (Figure 5B). In *vab-1* mutants levels of WVE-1 decrease and more WVE-1 is released from membranes (Figure 5B, 5D).

### Post-embryonic genetic interactions of WAVE/SCAR genes and axonal guidance genes during neuronal migrations

To explore how the three guidance pathways might signal to WAVE/SCAR during post-embryonic neuronal cell migrations, we took advantage of gain-of-function alleles in each guidance receptor. These alleles have been used to ask if other genes are genetically downstream of these guidance signals in neurons. CED-10/Rac1 has been placed genetically downstream of UNC-40 and of SLT-1/SAX-3 due to the ability of loss-of-function mutations in *ced-10/Rac1* to partially suppress gain-of-function mutations in SLT-1 and UNC-40 that alter the post-embryonic migrations of axons labeled with *mec-4::gfp (zdIs5*) [16], [39]. Further, similar gain-of-function studies of VAB-1/Eph using myristoylated VAB-1 have been used to identify genes, including NCK-1 and Wasp, that function downstream of VAB-1 [40]. We used these same strains to ask if WAVE/SCAR behaves like a component of the netrin, Robo or ephrin pathway during post-embryonic neuronal migrations.

#### WAVE/SCAR complex mutants affect axonal guidance of the AVM neuron

**Figure 6:**
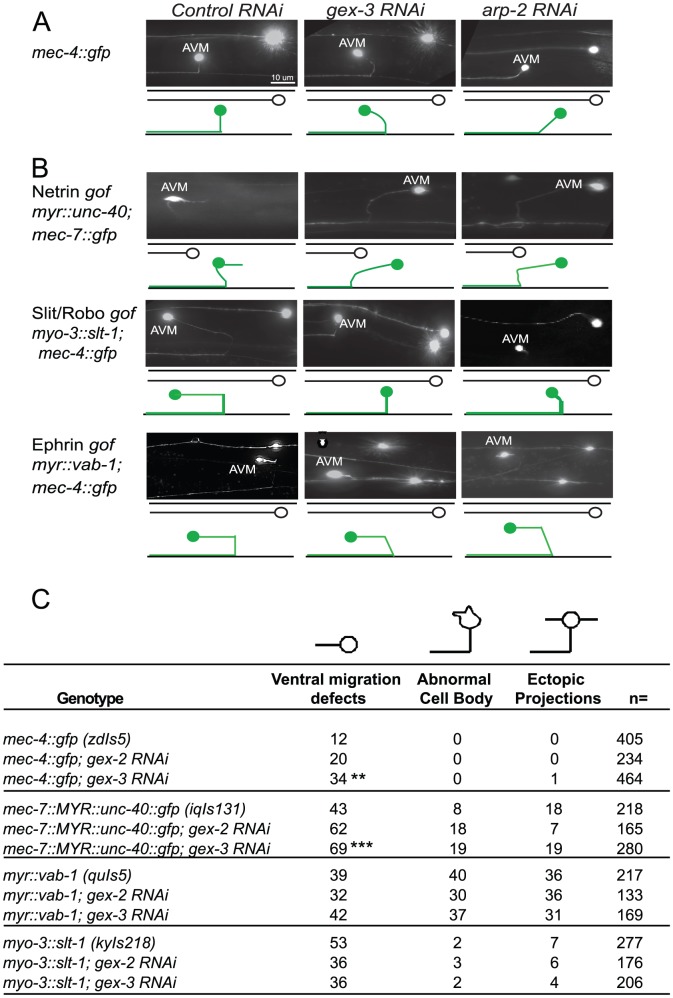
Post-embryonic genetic interactions of WAVE/SCAR genes and axonal guidance genes during neuronal migrations. (A) *WAVE/SCAR and Arp2/3 are required for ventral axonal guidance of the AVM neuron*. The *mec-4::gfp* (*zdIs5*) transgene is expressed in 6 mechanosensory neurons [41]. Micrographs show representative ventral migration patterns. The cartoons below each micrograph show the path of the AVM axon (green) with ALM (black) for reference. In wild type animals the AVM axon migrates ventrally, then anteriorly towards the head. Anterior is on the left. Control RNAi: nematodes were fed the RNAi empty vector, L4440 in HT115 bacteria. (B) *Loss of WAVE/SCAR or Arp2/3 partially suppresses the AVM ventral migration defect caused by misexpression of Slt/Robo*. The nematode strains *myo-3::slt-1 (kyIs218), myr::vab-1(quIs5)* and *myr::unc-40::gfp (lqIs131*), carry transgenes that cause *gain of function* phenotypes in three axonal guidance receptors. *gof* = gain-of-function. (C) Summary of AVM defects: ventral migration defects, abnormal cell bodies and ectopic projections. Animals were cultured at 20°C. Asterisks indicate significant change in the phenotypes compared to the single mutants. ** = p<.01, *** = p<0.001 as determined by a One-way Anova test followed by the Tukey test.

We first characterized the effects of WAVE/SCAR loss on the mechanosensory neuron reporter, *mec-4::gfp* [41], focusing on the well-characterized migration of the axon of the AVM motor neuron. In wild type animals carrying this transgene the AVM cell body is relatively round, it does not make extra projections, and it has one axon that migrates ventrally to the nerve cord and then anteriorly towards the head. 12% of the animals show minor perturbations in the ventral migration. Depletion of *gex-2* or *gex-3* via RNAi led to frequent defects in the ventralward migration of the axon (20–34%, compared to 12%, Figure 6A). In contrast, depletion of *gex-2* or *gex-3* did not result in significant effects on cell body shape and only occasionally caused ectopic projections (1%) (Figure 6C).

Animals with a transgene containing myristoylated UNC-40::GFP (*iqIs131*), a proposed gain-of-function strain, display a 43% penetrant defect in the ventral migration of AVM, 8% have AVM cell body abnormalities and 18% have ectopic projections. Depletion of *gex-2* or *gex-3* via RNAi did not suppress these phenotypes. Instead, the ventral guidance defect was enhanced to 69%, suggesting an additive effect.

Myristoylated VAB-1::GFP, a proposed gain-of-function transgene (*quIs5*), leads to a 39% penetrant defect in the ventral migration of AVM, as well as 40% penetrant defect in cell body abnormalities and 36% penetrance of ectopic projections. Depletion of *gex-2* or *gex-3* via RNAi did not suppress these phenotypes. Therefore, Myr-VAB-1::GFP may not require WAVE/SCAR for the aberrant AVM behaviors.

Expression of SLT-1 under the *myo-3* promoter (*kyIs218*) results in SLT-1 signal from both dorsal and ventral muscle cells, rather than just dorsal cells, which leads to SAX-3 activation from both ventral and dorsal regions. This *slt-1/sax-3 gain-of-function* results in a 53% penetrant defect in the ventral migration of AVM, with 2% cell body abnormalities and 7% ectopic projections. Depletion of *gex-2* or *gex-3* via RNAi led to partial suppression of the ventral migration defects, (to 36%) suggesting that SLT-1 and SAX-3/Robo may signal through WAVE/SCAR.

## Discussion

Our results suggest that the ventralward enrichment of F-actin is an essential event that sets up properly organized and dynamic actin protrusions that result in cell migrations during *C. elegans* morphogenesis. Models of WAVE/SCAR activation propose that receptor signaling leads to activated Rac (Rac-GTP) that can specifically bind to the GEX-2/Sra1/p140/PIR121/CYFIP component of the WAVE/SCAR complex [30]. Thus, Rac activation helps recruit the WAVE/SCAR complex to membranes where it can promote cellular protrusions or other Arp2/3-dependent events [29].

We show here that three signaling pathways regulate the actin cytoskeleton during embryonic development through differential effects on the actin regulators CED-10/Rac1 and WAVE-1. These studies suggest how multiple signals that assemble at cell membranes are combined to induce the polarized distribution of molecules that promote polarized distribution of F-actin.

### Why had netrin effects on embryonic morphogenesis been missed?

While the ephrin and slit/robo pathways had long been examined for embryonic morphogenesis effects, there was little notice of netrin embryonic roles. The one exception is the 1990 netrin pathway paper by Hedgecock and colleagues that reported embryonic neuronal and mesodermal cell migration defects [1]. We observed that all four *unc-40* putative null alleles and the *unc-6* deletion null showed low penetrance embryonic lethality, and a small but significant number of embryos with the Full Gex phenotype (no enclosure). This was true after we out-crossed four putative null *unc-40* mutants and the *unc-6* deletion null strain twice. Therefore it is highly unlikely that secondary mutations or strain background can explain the *unc-40* and *unc-6* embryonic phenotypes.

### Guidance receptors regulate embryonic F-actin dynamics

The distribution of F-actin in the migrating epidermal cells is highly dynamic and enriched toward the leading edge. We previously showed that loss of the WAVE complex leads to fewer, smaller protrusions at the leading edge of the migrating epidermal cells [15]. The movies made for this study show waves of F-actin enrichment that are dynamically oriented toward the leading edge (Video S1 and S2). *In vivo* imaging of F-actin distribution in embryos missing either the netrin, Robo or ephrin receptors demonstrated quantifiable changes in epidermal F-actin levels and enrichment. When the epidermal cells initiate their migrations, robust filopodial and lamellar protrusions extend from the two Leading Cells at the anterior epidermis. Loss of *sax-3* or *vab-1* caused a significant delay in Leading Cell protrusions that correlated with a drop in ventralward enrichment of F-actin (Figure 2A, 2E). These changes in F-actin distribution help explain the high percent of embryonic lethality seen in *sax-3* and *vab-1* embryos. In contrast, loss of *unc-40* permitted ventral protrusions in all embryos examined, and ventralward enrichment of F-actin, despite a drop in overall F-actin levels. This helps explain the much milder embryonic lethality seen in *unc-40* and *unc-6* mutant embryos and suggests F-actin polarization or enrichment is more important for cell migration than total F-actin levels.

Removal of any of the guidance cues analyzed here altered the rate of protrusion turnover (Figure 3, Video S3). Some actin regulators, like cofilin, are involved in the turnover of protrusions by helping to disassemble polymerized actin, and thus contribute to the recycling of actin components that promotes cell movements [42], [43]. Other actin regulators, like myosin II and Rho, help turn over protrusions by helping to remodel adhesive structures [44]. Turnover of protrusions is thus an essential component of cell migrations. We propose that axonal guidance cues may contribute to all of these kinds of actin changes that promote cell migrations including protrusion formation and turnover. More thorough understanding of how changes at the membrane result in dramatic changes in the F-actin levels and enrichment shown here will require additional studies. However, our identification of CED-10 and WVE-1 as essential read-outs of the signals is an important first step in understanding this complicated process.

### Model for the regulation of CED-10/Rac1 and WAVE/SCAR by the three guidance cues

**Figure 7:**
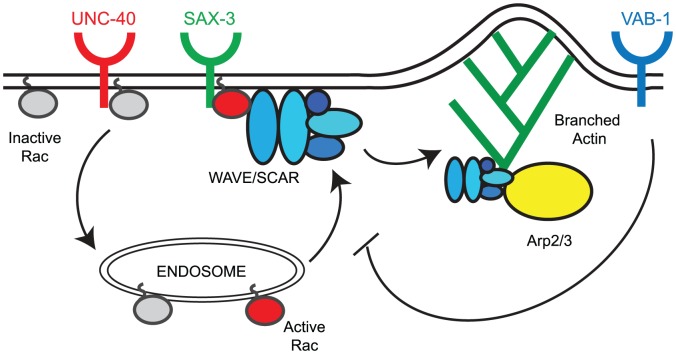
Model for the regulation of the CED-10/Rac1 – WAVE/SCAR – Arp2/3 pathway by axonal guidance receptors. UNC-40 activity supports the transport of inactive CED-10/Rac1 (Rac1-GDP, grey circles) to endosomes where it becomes activated (Rac1-GTP, red circles). Activated CED-10/Rac1 is then targeted to the plasma membrane where it is recruited by SAX-3/Robo. Activated CED-10/Rac1 can then recruit and help assemble the WAVE/SCAR complex (blue ovals), which leads to robust branched actin polymerization through the Arp2/3 complex (yellow oval). VAB-1/Ephrin inhibits the targeting of activated CED-10/Rac to the membrane thereby modulating branched actin polymerization.

Loss of *unc-40*, *sax-3* and *vab-1* altered the distribution and levels of CED-10::GFP and GFP::WVE-1 at subcellular regions. To understand how three distinct pathways could contribute to the correct regulation of cell movements that depend on actin regulators, we tested the model that all the pathways act redundantly. Genetic and molecular studies show that a simple redundancy model does not explain the integration of three signals to regulate the WAVE/SCAR actin nucleation complex. Instead, our results suggest the following Model for the roles of the three signaling pathways (Figure 7). We propose that UNC-40 activity supports the transport of inactive CED-10/Rac1 (Rac-GDP, grey circles in Figure 7) to endosomes where it is activated (Rac-GTP, red circles, Figure 7). Activated CED-10/Rac1 is then targeted to the plasma membrane where it is recruited by SAX-3/Robo. Activated CED-10/Rac1 can then recruit and help assemble the WAVE/SCAR complex, which leads to robust branched actin polymerization through the Arp2/3 complex. VAB-1/Ephrin inhibits the targeting of activated CED-10/Rac to the membrane thereby modulating branched actin polymerization.

#### Model for SAX-3/Robo recruitment of CED-10/Rac1 to promote embryonic morphogenesis

SLT-1/Robo signaling requires Rac downstream [22], [23]. The results shown here suggest a relatively simple relationship between SAX-3/Robo and CED-10/Rac1 during epidermal morphogenesis in *C. elegans*. SAX-3/Robo signaling helps to recruit CED-10/Rac1 to the membrane, which recruits the WAVE complex, and results in polarized nucleation of F-actin at the leading edge required for epidermal migrations.

#### Model for UNC-40/DCC activation of CED-10/Rac1 during embryonic morphogenesis

Netrin/DCC signaling has been shown to activate Rac1 *in vitro* [17]–[19] while genetic studies support a role for CED-10/Rac1 downstream of UNC-40/DCC [16]. We propose that the wild type function of UNC-40 is to promote CED-10 activation. Loss of UNC-40/DCC resulted in altered CED-10 and WVE-1 distribution, and decreased F-actin levels. Subcellular fractionation showed that while overall WVE-1 levels were slightly elevated, WVE-1 became highly enriched in the low speed pellet, suggesting a shift of WVE-1 to new membrane locations. In addition, CED-10 appeared more enriched basolaterally (Figure 4B), while WVE-1 was less enriched basolaterally (Figure 5B). We interpret this to mean that in *unc-40* mutants CED-10 at this apparent basolateral location is less capable of recruiting WVE-1 appropriately. Palamidessi and colleagues found that Rac1 requires trafficking to endosomes to become active at the membrane [45]. We propose that UNC-40 is involved in activating CED-10/Rac1 by ensuring it is appropriately trafficked to the correct endosomes. Localization studies with antibodies to active and inactive *C. elegans* CED-10/Rac1, which are not currently available, could test this model. In addition, it may be possible to use antibodies to activated mammalian Rac1 to immunoprecipitate active CED-10 in wild type lysates and in lysates depleted of UNC-40 to test this model.

Netrin effects on morphogenesis are quite mild (Figure 1, Table 1), in spite of the changes in CED-10 and WVE-1 localization (Figure 4, Figure 5), and decreased F-actin levels (Figure 2). The mildness might be explained by partial redundancy with other signaling pathways including SAX-3/Robo. The *unc-40; sax-3* double showed a synergistic increase in the Full Gex morphogenesis phenotype which could indicate redundancy. In the absence of UNC-40, SAX-3 continues to recruit (and possibly activate) CED-10 at the membrane, even without UNC-40 activation of CED-10 (Figure 1C, Table 1). An alternative way to explain the mild netrin phenotypes is that the higher amounts of CED-10 at membranes induced by *unc-40* loss, even if not fully polarized, result in enough CED-10 and WVE-1 activity that is able to assemble polarized F-actin to promote ventral protrusions.

#### Model for VAB-1/Ephrin Receptor modulation of CED-10/Rac1 to promote embryonic morphogenesis

In *vab-1* mutants CED-10 appears more enriched at basolateral regions (Figure 4B), but WVE-1 appears less enriched at basolateral regions (Figure 5B). Subcellular fractionations show decreased WVE-1 in the higher speed pellets, suggesting a shift of WVE-1 away from some membranes. This results in high but disorganized F-actin levels (Figure 2). These results are distinct from the changes seen in *sax-3* or *unc-40* mutants. In neurons, activated ephrin receptor is proposed to negatively regulate Rac1 [15]. Given the role of ephrins in membrane and cytoskeletal remodeling during synaptic transmission, one attractive possibility is that ephrin signaling regulates the abundance of guidance receptors at the membrane that is required for F-actin reorganization in the epidermis. Since more F-actin is nucleated in *vab-1* mutants, and protrusion turnover is delayed (Figure 3) one potential role for VAB-1 is to negatively regulate CED-10 activation or localization. If the turnover of CED-10 and its associated receptors decreases at the membrane, this could contribute to the results we see: ectopic F-actin polymerization in less dynamic cells, decreased ventralward enrichment of F-actin, and delays in cell migration. Similar experiments to those proposed for testing the role of UNC-40 could test the model that there is extra CED-10 activation, but at the wrong place, in *vab-1* mutants. Alternatively, the increased F-actin seen in *vab-1* mutants may be due to up-regulation of another pathway that promotes actin nucleation when VAB-1 is missing. For example, VAB-1 has a mutually inhibitory interaction with the PIP3 Phosphatase PTEN/DAF-18 [46], and PTEN/DAF-18 contributes to embryonic development [47].

### Where does VAB-1 act during embryonic epidermal cell migrations?

VAB-2 and VAB-1, Ephrin and Ephrin receptor, were thought to act in the underlying neuroblasts for epidermal morphogenesis to occur [6]–[8]. However recent studies of the *vab-1* promoter fused to GFP suggest that some epidermal cells express VAB-1 [48]. More fine-tuned analysis of actin dynamics in the epidermal and neuronal cells in animals depleted of ephrin and ephrin receptor could address the tissue requirements of ephrins during this process.

### Do the interactions shown here extend to neurons?

WAVE/SCAR components have been shown to affect axonal guidance and ectopic branching in the PDE neuron [14]. Loss of WAVE/SCAR components affected the ventral migration of the AVM axon (Figure 6A), suggesting WAVE/SCAR proteins contribute to some of the same axonal guidance events that are regulated by netrins, slt-1/Robo and ephrins. One caveat is that we depleted the WAVE/SCAR components by RNAi in all tissues, so these effects on neurons could also be due to the functions of WAVE/SCAR in other tissues. Tissue-specific depletion of WAVE/SCAR components would clarify this issue. We tested if the role of WAVE/SCAR downstream of axonal guidance receptors extends to neurons by using gain-of-function strains that alter the AVM axonal migration (Figure 6). The ability of WAVE/SCAR and Arp2/3 depletion to suppress the *slt-1/sax-3* gain-of-function defects may support our Model that SLT-1/Robo can signal through Rac, and that Rac activates WAVE/SCAR. The inability of WAVE/SCAR to suppress the Myr-VAB-1 gain-of-function defects suggests that Ephrin in *C. elegans* has targets other than WAVE/SCAR. For example, it has been suggested that VAB-1 signals through Wasp and NCK-1 [40]. The inability of WAVE/SCAR depletion to suppress Myr-UNC-40 phenotypes was a surprise, given the expectation that UNC-40 activates Rac, which can activate WAVE/SCAR. There are several ways to interpret this result. Myr-UNC-40::GFP may alter other cytoskeletal components other than the WAVE/SCAR complex to interfere with the correct AVM axon migration. Another possibility is that Myr-UNC-40, which is retained at the membrane, may not be simply more active. If the failure of Myr-UNC-40 to turn over, or to interact with other UNC-40 Receptors blocks a positive cue, then Myr-UNC-40 may be causing a *loss-of-function* phenotype. In relation to our proposed model, if UNC-40 activation of CED-10/Rac1 supports SAX-3 recruitment of active CED-10/Rac1, then UNC-40 loss would enhance defects caused by loss of WVE components, just like it enhances loss of SAX-3. An alternative interpretation is that in the absence of WAVE/SCAR in all tissues (as we did here) other axonal molecules, like SLT-1, are not properly localized. This could lead to enhancement of the Myr::UNC-40 defects.

Future experiments with reagents that can show how loss of one signal affects the other signals will be helpful in further determining how these multiple signals converge to regulate CED-10/Rac1 enrichment at membranes, WAVE/SCAR recruitment and activation, and properly polarized and dynamic F-actin.

## Materials and Methods

### Strains

The following strains were used in this analysis: *unc-40(n324), unc-40(e1430), unc-40(n473), unc-40(e271), unc-6(ev400), sax-3(ky123), sax-3(ky200), vab-1(dx31), vab-1 (e2), FT48 xnIs16 [dlg-1::gfp]; him-8, OX469 unc-40(n324); xnIs16 [dlg-1::gfp], OX248 unc-40(e1430); xnIs16 [dlg-1::gfp]), OX243 unc-6(ev400); xnIs16 [dlg-1::gfp], OX242 sax-3(ky123); xnIs16 [dlg-1::gfp], OX485 vab-1(dx31); xnIs16 [dlg-1::gfp], OX471 pjIs1[gfp::wve-1; rol-6], OX490 unc-40(n324); pjIs1 [gfp::wve-1; rol-6], OX491 sax-3(ky123); pjIs1 [gfp::wve-1; rol-6], OX484 vab-1(dx31); pjIs1 [gfp::wve-1; rol-6], OX466 pjIs4 [ced-10::gfp::ced-10; unc-76 (+)], OX482 unc-40(n324); pjIs4 [ced-10::gfp::ced-10], OX480 sax-3(ky123); pjIs4 [ced-10::gfp::ced-10], OX483 vab-1(dx31); pjIs4 [ced-10::gfp::ced-10], ML1154 mcIs51 [plin26::vab-10ABD::gfp; pmyo-2::gfp], OX353 gex-3(zu196)/Dnt1;mcIs51 [plin26::vab-10ABD::gfp; pmyo-2::gfp], OX487 unc-40(n324); mcIs51 [plin26::vab-10ABD::gfp; pmyo-2::gfp], OX521 unc-6(ev400); mcIs51 [plin26::vab-10ABD::gfp; pmyo-2::gfp], OX488 sax-3(ky123); mcIs51 [plin26::vab-10ABD::gfp; pmyo-2::gfp], OX489 vab-1(dx31); mcIs51 [plin26::vab-10ABD::gfp; pmyo-2::gfp], OX441 unc-40(n324)/hT2; vab-1(dx31), OX134 unc-40(e1430); sax-3(ky200), IC361 vab-1(e2)/mIn1; sax-3 (ky123), SK4005 zdIs5 [mec-4::gfp], LE2661 lqIs18 [mec-7::myr unc-40::gfp], CX5078 dpy-20(e1282)IV;zdIs5; kyIs218 [myo-3::slt-1/40 integrated+str-1::gfp+dpy-20(+)], IC400 zdls5 I;quls5 [myr-vab-1] II;him-5(e1490)V.*

### Lysates for biochemistry

#### Whole-worm lysates

(Figure 4C, Figure 5C) To look at the change in total levels of proteins, small scale lysates were prepared by boiling 100 adult worms in 40 µl of RIPA buffer (15 mM HEPES, 140 mM NaCl, 3 mM MgCl2, 1 mM EDTA, 0.5% Sodium Deoxycholate, 1% NP-40, 2% SDS) with 20 µl 5× Laemmli sample buffer. These lysates contain both supernatant and pellet components and are therefore termed whole worm lysates. 15 ul of each lysate (equivalent to approximately 25 worms) were loaded on a 10% SDS-PAGE gel.

#### Embryonic lysates

(Figure 5C) were prepared as in [12]: Briefly, large populations of worms were collected and the adults were dissolved using a hypochlorite solution to collect the embryos. Embryos were homogenized in Lysis Buffer (150 mM NaCl, 1 mM DTT, 0.1% Triton and protease inhibitor cocktail - Roche #11836153001) in a stainless steel homogenizer (Wheaton #357572). Lysates were spun at 5 K for 5 minutes to separate the supernatant from the pellet. The pellet fraction was resuspended in 100 ul of Lysis Buffer. Both supernatant and pellet were diluted to a concentration of 1 ug/ml and 10 ug in total were loaded on an SDS-PAGE gel.

### Subcellular fractionations

Lysates were prepared as described for embryonic lysates above but mixed staged animals were used and Triton was excluded from the Lysis buffer. All lysates were processed immediately. The lysates were spun at increasing speeds and times using a Beckman Coulter Optima TLX Ultracentrifuge, rotor # TLA-120.2. The supernatant was removed after each spin and spun at the next speed. The spin speeds and times were: spin 1, 1 K rpm for 10 minutes; spin 2,10 K rpm for 10 minutes; spin 3, 30 K for 20 minutes; spin 4, 100 K for 90 minutes. At each step the pellet was resuspended in a volume equal to the supernatant it was derived from. 5× Laemmli Buffer was then added to each fraction, the fractions were boiled at 95°C for 5 minutes before loading on gels for Western blotting. Equal volumes of each supernatant and pellet pair were loaded per lane.

### Feeding RNAi

For all feeding RNAi, cDNAs of the genes were inserted into L4440 vector and transformed into HT115 cells. Saturated overnight cultures were diluted 1∶250 and incubated for 6–7 h at 37°C with agitation until the OD600 was close to 1. Bacteria were pelleted by centrifugation and resuspended in LB Amp, 100 µg/ml. 1 mM IPTG was added to the bacteria and Amp plates before use. *C. elegans* animals were synchronized by hypochlorite treatment followed by hatching in M9 Buffer. For lysates L1 worms were fed either control HT115 E. Coli, or HT115 containing the L4440 plasmid carrying the gene of interest and were grown at 20°C for 3 days. Lysates were made from the mixed population of adults and eggs. For embryonic analysis embryos were collected on the third day and imaged. For adult analysis, animals were assayed for neuronal phenotypes after two days on the RNAi food. All experiments were performed at 20°C unless otherwise noted.

### Immunostaining and microscopy of embryos

For immunohistochemistry, embryos were fixed to poly-lysine slides and freeze-cracked by incubating on dry ice for 15 minutes. Slides were then fixed in methanol for 20 minutes at −20°C, blocked in 2% PBST for 5 minutes, washed 3 times with PBS and incubated with primary antibodies for 1 hour at 37°C. Slides were then washed 3 times with PBS and incubated with secondary fluorophore conjugated antibodies for 1 hour at 37°C. Slides were mounted in PGND solution as described. All images (except live imaging of *plin-26::vab-10 ABD::gfp*, Figure 2, Figure 3) were acquired on a Zeiss Axioskop 2 Plus microscope using a 40× oil objective with iVision 4.0 software driving a Roper SensiCam QE camera. The images were then analyzed using ImageJ software.

### Live imaging of embryos

Two to four cell stage embryos (0–20 minutes after first cleavage) were dissected from adult hermaphrodites and mounted on 3% agarose pads in water. The embryos were incubated at 23°C for 240 minutes, then imaged every 2 minutes for at least 120 minutes. The interval was chosen to reduce photo bleaching of the signal and to avoid damage to the embryo due to the exposure to UV light.

### Laser spinning disc confocal microcopy

Live *plin-26::vab-10 ABD::gfp* embryos were imaged using a 40×1.3 NA oil immersion lens on a Nikon TE2000 inverted microscope fitted with a Yokogawa CSU21 spinning-disk confocal scanhead (Perkin Elmer), a Melles-Griot argon laser (514 nm excitation) controlled by a Neos programmable AOTF. Multidimensional datasets were acquired using the software MetaMorph on a Hamamatsu Orca-AG cooled CCD camera, and stereo QuickTimeVR movies were assembled from the raw data using a custom-written plug-in for the Java program ImageJ (http://rsb.info.nih.gov/ij/). Background noise from the camera was reduced to the lowest grey levels of the image bit depth, via a linear contrast stretch. Images taken at the time points of interest were analyzed using ImageJ software or the ImageJ-based GLOWormJ viewing and analysis program: http://www.glowormnotes.org.

### Quantitation of epidermal F-actin

Embryos expressing *plin26::vab10ActinBindingDomain::gfp* were filmed at 2-minute intervals. Stacks were projected into 4D QuickTime movies that allowed viewing of the embryos from multiple angles. All quantitation was done on the raw images. The figure legends indicate when images were enhanced for contrast, and the same enhancement was applied to a mosaic of the related images for that figure.

#### Figure 2C

To quantitate total levels of F-actin at the leading edge movies of live embryos were opened in the GLOWormJ program, which uses the ImageJ platform. *Timing:* measurements were done at the time of first F-actin enrichment at the leading edge of the Leading Cells (LCs). For wild type embryos this was ∼260 minutes after first cleavage. For *gex-3*, where actin is never recruited to the leading edge the measurements were taken at ∼260 minutes to match the wild type. For *unc-40*, *unc-6*, *sax-3*, and *vab-1* the measurements were taken at ∼270, ∼260, ∼275 and ∼270 minutes after first cleavage respectively. *Region measured*: the rectangle tool was used to draw a box, 36 by 36 pixels, that covered approximately a third of the length of the cell from the leading edge. A single reading of average fluorescence was taken for each embryo at the stated time point. The readings were then averaged and a One-way ANOVA test was used to test for significant changes in fluorescence levels. n for each genotype is shown in Figure 2C.

#### Figure 2D

To measure the distribution of F-actin in the LCs of the migrating epidermis along the dorsal/ventral axis, movies were analyzed using the GLOWormJ program. A single line was drawn through one of the LCs from the ventral to the dorsal side using the Line tool, and the intensity of the fluorescent signal was read along this line, and plotted. Areas of the cell with the highest fluorescent signal produced high readings, at least 10 fluorescent units higher than the background, which appeared as peaks on the plotted graph and were marked with an asterisk. Readings were taken for 20 frames, starting with the time point when actin was first enriched at the leading edge of the LCs, as described for Figure 2C. The distribution of the peaks on the graph was examined, either in the ventral half or in the dorsal half of the cell. Figure 2D shows one representative graph from the 20 that were generated per embryo examined.

#### Figure 2E

The number of peaks observed either in the ventral half or in the dorsal half were counted for 20 frames (40 minutes) during which the readings were taken. The total number of ventral peaks was divided by the total number of dorsal peaks for the 20 frames. This generated the ratio of ventral to dorsal actin enrichment. n for each genotype is shown in Figure 2E.

### Statistical analysis

All graphs show the mean of the data and the Standard Error of the Mean (SEM). For grouped data, statistical significance was established by performing a two-way Analysis of Variance (ANOVA) followed by the Bonferroni multiple comparison post-test. For ungrouped data a one-way ANOVA was performed followed by the Tukey post-test. Asterisks (*) denote p values<0.05. All statistical analysis was performed using GraphPad Prism.

## Supporting Information

Video S1 UNC-40/DCC, SAX-3/Robo and VAB-1/Eph are required for F-actin regulation during embryonic morphogenesis. Live *C. elegans* embryos of the indicated genotypes are shown during epidermal morphogenesis with F-actin marked by an epidermis-specific transgene, *plin-26::VAB-10 ABD::GFP (mcIs5)*. The movies, made at 23°C begin at the onset of epidermal migration (Figure 2B) and continue for approximately 40 minutes. The *unc-6* movie stops earlier than the others.
(MOV)

Video S2 Effects of UNC-40/DCC, SAX-3/Robo or VAB-1/Eph loss on the Leading Cells during morphogenesis. The two Leading Cells from an embryo of each genotype are highlighted to show the leading edge dynamics and ventral to dorsal F-actin enrichment in these cells during morphogenesis (same time points as Movie 1).
(MOV)

Video S3 Dynamics of protrusion turnover in wild type and morphogenesis defective mutants. Protrusion formation and turnover is illustrated using the *plin-26::VAB-10 ABD::GFP* transgene. The cells at the top of the frame at the beginning of the movies include the Leading Cells in the ventral row of epidermal cells that migrate ventrally (down) in wild type. The cells at the center of the frame in some of the movies are the dorsal epidermal cells, also labeled by this epidermis-specific transgene that visualizes F-actin.
(MOV)
